# Veterinary Register

**Published:** 1901-10

**Authors:** 


### VETERINARY REGISTER.

[See Editorial, Journal of Comparative Medicine and Veterinary Archives, July, 1901, page 438.]

[In the following list the names and data printed without comment are those furnished to us by postal card with the signature of the individual.

Those with an asterisk [*] are those to whom we have sent, by United States mail, an envelope with prepaid postage and a request for return if not delivered in ten days; as the envelopes have not been returned, we presume that the letter has been delivered, that the individual in question exists, but that he has neglected to return the enclosed postal card with the information requested.—Ed. Journal.]

### PENNSYLVANIA.

| *Name.* | *Street.* | *Town.* | *College, Graduated.* | *County, Registered.* |
|---|---|---|---|---|
| Cady, Thomas | ...... | Columbus | * | ...... |
| Cameron, Wm. D. | ...... | Philadelphia | * | ...... |
| Campbell, John B. | ...... | Washington | * | ...... |
| Campbell, S. M. | ...... | Pottstown | * | ...... |
| Carey, Charles R. | ...... | Carversville | * | ...... |
| Carnes, H. | Barney st | Wilkesbarre | * | ...... |
| Carr, David | ..... | Jamestown | * | ...... |
| Carter, E. | 167 Forbes st | Pittsburg | * | ...... |
| Carter, J. M. | 215 S. 17th st | Philadelphia | V. D. Univ. Pa., 1894 | Chester, 1894 |
| Cassel, Eli | 30 East Airy st | Norristown | ...... | Montgomery, 1887 |
| Cassidy, J. P. | ...... | Utica | * | ...... |
| Casterline, Warren | ...... | Wyoming | * | ...... |
| Cawley, Amos O. | 7 Arch st | Milton | American Vet., 1891 | Northumberland, 1891 |
| Cawley, W. W. | ...... | Lewisburg | * | ...... |
| Chamberlain, H. C. | Main st | Lanesboro | ...... | Susq'hanna, 1894 |
| Chrisman, M. J. | ...... | Sugar Grove | * | ...... |
| Christy, Henry S. | 2308 N. 15th st | Philadelphia | * | ...... |
| Christy, Joseph | ...... | Delmont | * | ...... |
| Church, D. S. | Care Ald'man Bailey | Scranton | * | .... |
| Church, H. R. | Main st | Luzerne | Ontario Vet., 1892 | Luzerne, 1892 |
| Clark, Robert B. | ...... | Fay | ...... | ...... |
| Clark, W. S. | 23 Barbour st | Bradford | Chicago Vet., 1892 | McKean, 1892 |
| Claypool, C. O. | ...... | Worthington | * | ...... |
| Cleaver, Kilburn H. | 14 N. Eighth st | Reading | Ontario Vet., 1879 | Berks, 1889 |
| Clemons, Holland | ...... | Covington | * | ...... |
| Clift, Charles H. | ...... | Mechanics Val. | * | ...... |
| Cline, William | 221 Queen st | Lancaster | * | ...... |
| Coble, Ira G. | Reagan av | Berwick | N. Y. Coll. V. S., 1893 | Schuylkill, 1889 |
| Cockley, Abraham | ...... | Penn | * | ...... |
| Coffman, Wm. B. | ...... | Frankstown | * | ...... |
| Coleman, David H. | Second st Pike | Philadelphia | * | ...... |
| Coles, W. H. | Main st | Mt. Pleasant | * | Westmoreland |
| Coles, M. S. | ...... | Stony Fork | * | ...... |
| Collins, C. O. | ...... | West Leesport | * | ...... |
| Collins, H. H. | ...... | Obolds | * | ...... |
| Collins, I. R. | ...... | Sharpsville | * | ...... |
| Collins, M. J. | ...... | Myerstown | * | ...... |
| Collins, Owen E. | ...... | Obold | * | ...... |
| Collner, Levi | ...... | St. Petersburg | ...... | Clarion, 1889 |
| Collum, Wm. B. | ...... | Doylestown | * | ...... |
| Comstock, J. R. | 5640 Penn ave | Pittsburg | * | ...... |
| Conard, M. E. | ...... | West Grove | * | ...... |
| Connolly, T. | ...... | Towanda | * | ...... |
| Conver, Aaron | ...... | Mariasville | * | ...... |

| *Name.* | *Street.* | *Town.* | *College, Graduated.* | *County, Registered.* |
|---|---|---|---|---|
| Cook, John | ...... | Emlenton | * | ...... |
| Cooke, Wm. A. | 2409 Master st | Philadelphia | ...... | Philadelphia, 1889 |
| Cooper, J. R. | ...... | Rural Valley | * | ...... |
| Corbett, Jas. W. | ...... | Pittsburg | * | ...... |
| Corbyn, Theop. N. | Tinicum and Island rds. | Philadelphia | ...... | Philadelphia, 1889 |
| Cornman, Ernest L. | 102 S. Beaver | York | V. D. Univ. Pa. 1900 | York, 1901 |
| Corson, Percy H. | ...... | Plymouth Meeting | * | ...... |
| Cottons, Samuel | ...... | Dawson | * | ...... |
| Countryman, G. F. | ...... | Lavansville | * | ...... |
| Courtright, John M. | ...... | Clarks Green | National Vet., 1895 | Lackawanna, 1895 |
| Cox, H. B. | 625 Wharton st | Philadelphia | American Vet., 1895 | Philadelphia, 1885 |
| Craft, Wm. S. | ...... | B'kwayville | ...... | Jefferson, 1889 |
| Crawford, Eli M. | Elmira st | Athens | Ontario Vet., 1891 | Bradford, 1891 |
| Crozier, Robert R. | ...... | Port Royal | ...... | ...... |
| Cropey, A. | ...... | Lawrenceville | * | ...... |
| Cullen, Charles M. | 762 Brooklyn | Philadelphia | V. D. Univ. Pa., 1887 | Philadelphia, 1889 |
| Culp, Francis | ...... | Arendtsville | * | ...... |
| Cummings, J. D. | ...... | Monroeton | * | ...... |
| Curtis, Chester E. | ...... | Nanticoke | * | ...... |
| Curtis, W. C. | ...... | Heart Lake | * | ...... |
| Czarneicki, J. H. A. | ...... | Allegheny | * | ...... |
| Czarneicki, C. I. | 43 First st | Allegheny | * | ...... |
| Czarneicki, E. L. | 43 First st | Allegheny | * | ...... |
| Dambach, Adam | ...... | Rochester | * | ...... |
| Davidheiser, A. K. | 126 King st | Pottstown | N. Y. C. V. S., 1892 | Montgomery, 1892 |
| Davies, M. A. | ...... | Troy | * | ...... |
| Davis, E. R. | ...... | Hatboro | ...... | Montgomery |
| Davis, I. Morris | ...... | Williams Cors. | * | ...... |
| Davis, J. F. | ...... | New Lebanon | * | ...... |
| Davis, Lewis R. | ...... | Hatboro | * | ...... |
| Davison, Richard Y. | Franklyn av | Morton | V. D. Univ. Pa., 1900 | Delaware, 1900 |
| Daynes, Edward | ...... | Somerset | * | ...... |
| Deardoff, J. W. | ..... | New Franklin | * | ...... |
| Deck, William | ...... | Bethel | * | ...... |
| Deckard, Israel K. | ...... | Middleton | * | ...... |
| Decker, G. W. | ...... | New Albany | * | ...... |
| Decker, Wilson S. | 607 Olive st | Scranton | * | ...... |
| Dehart, G. W. | 1158 Perkiom'n | Reading | * | ...... |
| De Haven, Frank R. | ...... | Wayne | * | ...... |
| Dengler, Harry O. | Main st | Norristown | Ontario Vet., 1888 | Montgomery, 1889 |
| Dennish, David B. | ...... | Morrisville | * | ...... |
| Denton, W. J. | 1820 Market st | Philadelphia | * | ...... |
| De Swan, George | 120 Robeson st | Myk., Phila. | * | ...... |
| Detar, Clem. M. | 25 W. Sixth st | Oil City | Ontario Vet., 1894 | Clarion, 1894 |
| Detrick, James M. | ...... | Stroudsburg | * | ...... |
| Detwiler, C. H. | ...... | Iron Bridge | * | ...... |
| Detwiler, D. C. | ...... | Iron Bridge | * | ...... |
| Detwiler, Geo. D. | ...... | Waterside | ...... | Bedford, 1889 |
| Devlin, C. F. | 2409 E. York st | Philadelphia | * | ...... |
| De Winney, Chas. | ...... | Codorus | * | ...... |
| Deipser, F. S. | ...... | Boyertown | * | ...... |
| Dickinson, W. M. | ...... | Philadelphia | * | ...... |
| Diemer, A. | ...... | Spring City | * | ...... |
| Dille, John N. | Main st | Prosperity | ...... | Washington, 1883 |
| Dilling, Jacob L. | ...... | Clover Creek | * | ...... |
| Dillwig, C. B. | ...... | Altoona | * | ...... |
| Dillwig, Geo. A. | ...... | Curryville | * | ...... |
| Dohan, Chas. A. | ...... | Darling | V. D. Univ. Pa., 1890 | Delaware, 1890 |
| Donmoyer, Edw. | ...... | E. Hanover | * | ...... |
| Donnellan, John J. | 30 High st | Pittston | ...... | Luzerne, 1895 |
| Dooley, Thos. | 306 Jefferson st | Philadelphia | * | ...... |
| Doorley, Thos. | 306 Jefferson st | Philadelphia | * | ...... |
| Doris, John J. | 815 Forbes st | Pittsburg | * | ...... |
| Dorney, Albert H. | 714 Hamilton | Allentown | * | ...... |

| *Name.* | *Street.* | *Town.* | *College, Graduated.* | *County, Registered.* |
|---|---|---|---|---|
| Downs, M. M. | 18 W. Minor st | West Chester | N. Y. C. V. S., 1891 | Chester, 1891 |
| Drake, Asher M., | ...... | Saltillo | * | ...... |
| Drake, M. W. | 1315 Wharton | Philadelphia | * | ...... |
| Drake, N. M. | 1315 Wharton | Philadelphia | * | ...... |
| Dreibelbis, P. K. | ...... | Dreibelbis Station | * | ...... |
| Drummond, G. G. | 2642 Gtn. ave | Philadelphia | * | ...... |
| Dry, Jas. B. | ...... | Bowers Sta'n | * | ...... |
| Dubbs, J. Elmer | ...... | Green Mount | * | ...... |
| Dubbs, W. G. | ...... | Fairfield | * | ...... |
| DuBois, G. B. | ...... | Wilkesbarre | * | ...... |
| Dubson, Jas. | ...... | Blandon | * | ...... |
| Duke, C. A. | 814 Green st | Altoona | * | ...... |
| Duncan, J. E. | ...... | Pittsburg | * | ...... |
| Dunlap, Geo. W. | 30 Church st | Ephrata | Ontario Vet., 1893 | Lancaster and Berks, 1893 |
| Dunlevy, Joshua L. | Pike st | Canonsburg | * | Washington, 1889 |
| Dunn, Geo. H. | 733 Arken ave | Pittsburg | * | ...... |
| Dunn, Harry E. | ...... | ...... | * | ...... |
| Dunn, R. A. | 18 W. Cent'l av | Titusville | American Vet., 1891 | Crawford, 1898 |
| Dunn, Wm. | ...... | Elliotsburg | * | ...... |
| Earnest, Charles H. | 2038 Wishart st | Philadelphia | * | ...... |
| Ebert, Reuben | ...... | Kempton | * | ...... |
| Echternacht, Henry | ...... | Adamstown | * | ...... |
| Eckman, T. T. | 142 N. Broad st | Philadelphia | * | ...... |
| Edwards, Warren T. | ...... | Downingtown | * | ...... |
| Elviage, J. C. | 1115 Peach st | Erie | Ontario Vet., 1890 | Mercer, 1890 |
| Emery, Harry | 606 Ross av | Wilkinsburg | Ohio Vet. Med., 1891 | Clarion, 1891 |
| Engle, Samuel S. | ...... | Murrell | * | ...... |
| Ensign, A. C. | ...... | Waymart | * | ...... |
| Entriken, Harry D. | 133 E. State st | Kennett Square | * | ...... |
| Erb, Daniel B. | ...... | E. Petersburg | * | ...... |
| Erthy, Enos | ...... | Walker | * | ...... |
| Erwin, W. E. | ...... | Somerton | * | ...... |
| Eshenbaugh, Lewis | ...... | Shiremanst'n | * | ...... |
| Eshleman, John M. | Strasburg av | Parkersburg | V. D. U. Pa., 1890 | Chester, 1890 |
| Ettinger, J. A. | Second st | Alburtis | ...... | Lehigh, 1884 |
| Evans, M. L. | ...... | Phoenixville | * | ...... |
| Fairly, James | 1511 Carpenter | Philadelphia | * | ...... |
| Fairve, Alex. | ...... | Latrobe | * | ...... |
| Fairve, Charles | 2433 Fairmount | Philadelphia | * | ...... |
| Fallows, George T. | 1734 Upland st | Chester | * | ...... |
| Fairley, John Henry | ...... | Johnstown | * | ...... |
| Faseninger, Joseph | ...... | New Bethlehem | * | ...... |
| Fatzinger, Chas. A. | 435 N. 8th st | Allentown | American Vet., 1890 | Lehigh |
| Faughman, J. C. | ...... | Mahanoy City | Ontario Vet., 1891 | Lycoming, Schuylkill, 1891 |
| Fay, James | 1326 Mt. Vernon | Philadelphia | * | ...... |
| Feather, Jacob | 11th st | Lebanon | * | Lancaster, 1889 |
| Feiser, R. P. | Main st | E. Berlin | Ontario Vet. | Adams, 1889 |
| Fellows, G. T. | 1134 Upland st | Chester | * | ...... |
| Felton, George | 138 Richmond | Philadelphia | * | ...... |
| Felton, H. B. | 934 Diamond | Olney | V. D. U. Pa., 1888 | Philadelphia, 1889 |
| Fergerson, Edwin | ...... | Meeker | * | ...... |
| Fergerson, J. R. | 1113 Peach st | Erie | * | ...... |
| Ferley, J. T., Jr. | 1431 S. 6th st | Philadelphia | American Vet., 1892 | Philadelphia, 1892 |
| Fernsler, Philip B. | 621 Chestnut st | Lebanon | N. Y. C. V. S., 1874 | Lebanon, 1889 |
| Fetherolf, G. R. | 209½ E. Center | Mahanoy City | * | ...... |
| Fetter, Henry R. | ...... | Brunnerville | * | ...... |
| Fetzer, J. M. | Main st | Coopersburg | Ontario Vet., 1886 | Lehigh, 1889 |
| File, George | ...... | Mars | Claims to only castrate. | |
| Fleiner, Christian | ...... | Mt. Oliver | * | ...... |
| Fletcher, Wm. M. | ...... | Greenville | ...... | Mercer, 1889 |
| Flood, E. H. | 2042 Diamond | Philadelphia | * | ...... |
| Fly, Joseph C. | 1243 N. 12th st | Philadelphia | ...... | Philadelphia, 1889 |
| Foelker, John C. | 119 S. 7th st | Allentown | ...... | Lehigh, 1889 |

| *Name.* | *Street.* | *Town.* | *College, Graduated.* | *County, Registered.* |
|---|---|---|---|---|
| Foelker, Samuel J. | 119 S. 7th st | Allentown | Ontario Vet , 1879 | Lehigh, 1889 |
| Folk, Simon S. | ...... | Elk Lick | * | ...... |
| Fooney, George | ...... | Silverspring | * | ...... |
| Foos, A. C. | Spruce and Laurel sts | Hazleton | Ontario Vet., 1887 | Luzerne, 1889 |
| Ford, Simon | ...... | E. Freedom | * | ...... |
| Formad, Robert | 1008 N. 6th st | Philadelphia | * | ...... |
| Fouse, C. E. | 16th & Poplar | Philadelphia | * | ...... |
| Fowler, Wm. H. | E. W'moreland & N. Front | Philadelphia | * | ...... |
| Fox, Philip M. | ...... | Burma | ...... | Clarion, 1890 |
| Fox, R. J. | ...... | Bryn Mawr | * | ...... |
| Fraker, Andrew J. | ...... | Scotland | * | ...... |
| Frantz, C. M. | 1104 Jackson st | Philadelphia | * | ...... |
| Frays, James D. | ...... | Hahnstown | * | ...... |
| Frazer, David L. | ...... | Lone Pine | * | ...... |
| Frazier, John | 131 W. Jefferson | Butler | * | ...... |
| Frazier, Wm. | 1219 Liberty st | | * | ...... |
| Frederici, U. G. | ...... | Tamaqua | * | ...... |
| Freed, Byron | ...... | Sharon | * | ...... |
| Freed, M. P. | ...... | Sharon | * | ..... |
| Fretz, John B. | ...... | Quakertown | * | ...... |
| Freund, Aug. | 152 E. 8th st | Bloomsburg | * | ...... |
| Frexler, J. P. | ...... | Shamokin Dam | * | ...... |
| Fried, Jacob J. | ...... | Stonybrook | * | ...... |
| Friedline, Jacob P. | ...... | Bakersville | * | ...... |
| Fritz, Simon P. | ...... | Pinehill | * | ...... |
| Fritzinger, Chas. H. | ..... | Penn | * | ...... |
| Frost, Benj. C. | ...... | Blossburg | * | ...... |
| Frutchy, E. | ...... | Portland | * | ...... |
| Fry, Hiram P. | ...... | Lititz | * | ...... |
| Fry, Wm. H. | ...... | Pine G've Mills | * | Centre, 1893 |
| Fuller, Geo. S. | 914 Brown st | Philadelphia | N. Y. C. V. S., 1890 | Philadelphia, 1890 |
| Fuller, W. N. | ...... | Philadelphia | * | ...... |
| Fulmer, S. | ...... | Alverton | * | ...... |
| Gable, A. E. | ...... | Lehighton | * | ...... |
| Gable, E. E. | ...... | Meadville | * | ...... |
| Gallagher, Michael | ...... | Pittsburg | * | ...... |
| Gallup, Chas. C. | ...... | Smethport | * | ...... |
| Garhard, Jonas K. | ...... | Hosensdack | * | ...... |
| Garrett, Casper | ...... | Lansdowne | * | ...... |
| Gatchell, E. M. | Box 2038 | West Chester | * | ...... |
| Gavin, Michael | 2429 Pine st | Philadelphia | ...... | ...... |
| Gay, J. S. | ...... | Terrytown | * | ...... |
| Gearhart D. C. | 206 Frankst'n | Pittsburg | American Vet., 1891 | Allegheny, 1891 |
| Gelbert, Chas. S. Jr. | 520 Spruce st | Scranton | V. D. U. of Pa., 1899 | Lackawanna, 1899 |
| Gentzell, D. E. | ...... | Spring Mills | ...... | Centre |
| George, H. F. | ...... | Upton | * | ...... |
| Gerberich, D. T. | ...... | E. Hanover | * | ...... |
| Gernert, Mathias | ...... | Ono | * | ...... |
| Gesner, G. W. | Upl'd & 71st sts | Philadelphia | * | ...... |
| Gibson, John J. | ...... | Cochranville | * | ...... |
| Gilbert, Ellwood G. | 112 High st | Pottstown | American Vet., 1884 | Montgomery, 1897 |
| Gillmore, Geo. B. | ...... | Pittsburg | * | ...... |
| Gise, J. B. | ...... | Gratz | ...... | Schuylkill, 1890 |
| Gish, Jacob M. | ...... | Newport | * | ...... |
| Gladfelter, M. H. | ...... | Springfield | * | ...... |
| Gladfelter, Robert | 1230 N. 18th st | Philadelphia | ...... | Philadelphia, 1889 |
| Glasgow Theo. | 800 N. 7th st | ...... | * | ...... |
| Glass, Alex. | 2125 Sansom st | Philadelphia | * | ...... |
| Goentner, Chas. T. | ...... | Bryn Mawr | American Vet., 1881 | Montgomery, 1891 |
| Goheen, Jas. | ...... | Fogelsville | * | ...... |
| Good, Chas. R. | 70 E. Church st | Lock Haven | Ontario Vet., 1887 | Clinton, 1889 |
| Gordner, Daniel R. | Water st | Hughesville | * | Columbia, 1889<br>Lycoming, 1894 |
| Gorman, Chas. H. | 1012 S. 25th st | Philadelphia | * | Philadelphia, 1889 |
| Gorman, D. A. | ...... | Hortons | * | ...... |
| Goss, Wilson | ...... | Montgomery | * | ...... |

| *Name.* | *Street.* | *Town.* | *College, Graduated.* | *County, Registered.* |
|---|---|---|---|---|
| Gould, Edward | ...... | Mt Bethel | * | ...... |
| Graham, Jas. | 112 Queen st | G't'n, Phila. | Royal Edinburgh (Dick) Vet., 1886 | Philadelphia |
| Gramley, J, A. | Main st | Mifflinburg | ...... | Centre 1889 |
| Green, Wm. B. | ...... | Greencastle | * | ...... |
| Greensweight, Wm. | ...... | Williamsport | * | ...... |
| Gregan, J. H. | ...... | Danville | * | ...... |
| Gregg, Ira M. | ...... | Garwood | * | ...... |
| Gregory, J. H. | ...... | Danville | * | ...... |
| Gregory, Sidney | ...... | Laceyville | * | ...... |
| Gregory, Wm. | ...... | Castile | * | ...... |
| Greth, Peter | 2061 E Som'rs't | Philadelphia | * | ...... |
| Gridley, C. M. | ...... | Ulysses | * | ...... |
| Griest, Franklin W. | ...... | Osceola | * | ...... |
| Griger, Johan | ...... | Plymouth | * | ...... |
| Grinniss, Geo. | 184 S. Wash't'n | Wilkesbarre | * | ...... |
| Griton, Jacob L. | ...... | Bloomsburg | * | ...... |
| Grofi, Benj. F. | 128 N. King st | Lancaster | * | ...... |
| Groff, Elias, | ...... | Bernville | * | ...... |
| Gronan, Jas. | ...... | Philadelphia | * | ...... |
| Groner, Lewis R. | Main st | Bath | Ontario Vet. 1886 | Northampton, 1889 |
| Gross, Andrew B. | ...... | Lurgan | * | ...... |
| Griebe, Andrew | ...... | Carlisle | * | ...... |
| Gruber, C. D. | ...... | Obold | * | ...... |
| Gruver, Elias A. | ...... | Richl'ndt'wn | N. Y. C. V. S., 1894 | Bucks, 1894 |
| Haas, Charles | ...... | Fogelsville | * | ...... |
| Haas, James A. | 120 Locust st | Harrisburg | American Vet., 1892 | Lehigh, 1889 |
| Haas, Solomon | ...... | Lyon Valley | * | ...... |
| Haber, Josiah K. | ...... | Tylersport | * | ...... |
| Hackler, H. D. | 2101 Amber st | Philadelphia | * | ...... |
| Hadley, John F. | 1235 S. 15th st | Philadelphia | * | ...... |
| Hagenbuch, J. B. | 5050 Balt'e av | Philadelphia | V. D. U. Pa., 1895 | Philadelphia, 1895 |
| Haley, Peter | ...... | Shenandoah | * | ...... |
| Haley, P. | N. W. cor. 22d and Vine sts | Philadelphia | * | ...... |
| Hallet, Wm. R. | ...... | Millport | * | ...... |
| Hamilton, F. A. | 136 W. Long av | Dubois | Ohio Vet. Med., 1895 | Clearfield, 1895 |
| Hamilton, F. A. | ...... | Maple Creek | * | ...... |
| Hamme, J. H. | 715 W. Poplar | York | American Vet., '89-90 | York, 1890 |
| Hammerschmidt, J. | 1033 N. 3d st | Philadelphia | * | ...... |
| Haney, Felix W. | ...... | Tannersville | ...... | Monroe, 1888 |
| Hanns, Robert | 3906 Terrace st | Wissa'n, Phila. | * | ...... |
| Harbison, John D. | ...... | Saxonburg | * | ...... |
| Harding, Richard | ...... | Penn | * | ...... |
| Harger, S. J. J. | 205 N. 20th st | Philadelphia | * | ...... |
| Harley, John | ...... | Hemlock | * | ...... |
| Harman, John A. | ...... | Stahlstown | ...... | W'moreland, 1889 |
| Harper, David S. | ...... | Susquehanna | * | ...... |
| Harper, M. D. | Box 77 | Breinigsville | N. Y. C. V. S., 1896 | Lehigh, 1898 |
| Harrigan, J. H. | 26th & Federal | Philadelphia | * | ...... |
| Harris, A. E. | ...... | Coal Centre | ...... | Washington, 1892 |
| Harris, David B. | ...... | Cisnarun | * | ...... |
| Harris, Milton | 1603 Darien st | Philadelphia | * | ...... |
| Hart, Walter L. | 2577 Amber st | Philadelphia | * | ...... |
| Hartman, G. R. | 934 Diamond st | Philadelphia | V. D. U. Pa., 1888 | Philadelphia, 1889 |
| Hastings. George S. | ...... | Tidioute | * | ...... |
| Haverly, Alex. C. | ...... | Overton | * | ...... |
| Haverly, Wm. D. | ...... | Campbellville | ...... | Sullivan, 1889 |
| Heaton, R. D. | ...... | Ashland | * | ...... |
| Heberton, C. M. | ...... | Hughesville | * | ...... |
| Heckenberger, H. | 120 Broad st | Pittston | * | ...... |
| Heckenberger, J. | 110 Front st | Catasauqua | * | ...... |
| Heckenberger, M.W. | Upper Main st | Slatington | * | ...... |
| Heckenberger, W. A. | 112 Front st | Catasauqua | * | ...... |
| Hefner, Wm. | 211 Queen st | York | * | ...... |
| Heffner, Benj. Y. | ...... | Aula | * | ...... |
| Heffner, W. H. | ...... | York | * | ...... |
| Hepfer, Danl. J. | ...... | Aula | * | Franklin, 1889 |

| *Name.* | *Street.* | *Town.* | *College, Graduated.* | *County, Registered.* |
|---|---|---|---|---|
| Heiser, Edward | ...... | Pottsville | * | ...... |
| Helmer, Jacob, | 311 Spruce st | Scranton | * | ...... |
| Helmer, R. C. | 311 Spruce st | Scranton | * | ...... |
| Helmly, Jacob R. | ...... | Durlach | * | ...... |
| Hendren, S. G. | ...... | Lewistown | * | ...... |
| Henrich, Abraham | ...... | New Berlinville | * | ...... |
| Henry, Calvin B. | ...... | Sigel | * | ...... |
| Henry, Thos. E. | ...... | Scotch Hill | * | ...... |
| Hepler, Isaac | ...... | Oakland | * | ...... |
| Herbst, David S. | ...... | Springfield | * | ...... |
| Herbst, W. S. | ...... | Springfield | * | ...... |
| Hershey, H. H. | ...... | Intercourse | * | ...... |
| Hetrick, Nicholas | ...... | Bodines | * | ...... |
| Hewitt, Fred S. | 201 Luzerne av | Pittston | * | ...... |
| Hewitt, Thos. | ...... | Frazer | * | ...... |
| Heyler, Gottlieb | ...... | Sebring | * | ...... |
| Hibbard, H. C. | ...... | Bernice | * | ...... |
| Hibbs, J. S. | ...... | Fallsington | * | ...... |
| Hickman, G. L. K. | 145 N. Front st | Steelton | * | ...... |
| Hickey, Albert | ...... | Middlesex | * | ...... |
| Hickey, Windle, | ...... | Harmony | * | ...... |
| Hill, I. C. | ...... | Jackson | * | ...... |
| Hillegas, Jesse F. | ...... | Red Hill | Ontario Vet., 1890 | Montgomery, 1890 |
| Hinman, Albert W. | 614 4th st | Braddock | * | ...... |
| Hodge, Isaac | ...... | Monongahela | * | ...... |
| Hoffman, F. F. | Box 105 | Brookville | Ontario Vet., 1885 | Jefferson |
| Hoffman, John P. | ...... | Gettysburg | * | ...... |
| Hoffman, Sol. K. | ...... | Hamburg | Ontario Vet., 1884 | Berks, 1889 |
| Hogg, Edwin | 11 N. Canal st | Wilkesbarre | V. D. U. Pa., 1895 | Lancaster, 1895 |
| Holler, Harrison K. | ...... | Terehill | * | ...... |
| Holley, Charles W. | ...... | Muncy | * | ...... |
| Holmes, E. B. | Second av | Royersford | Ontario Vet., 1891 | Montgomery, 1892 |
| Holt, F. F. | 116 N. 7th st | Philadelphia | * | ...... |
| Hoovel, John C. | ...... | Tionesta | * | ...... |
| Hoover, Benj. F. | ...... | Grahamton | * | ...... |
| Hoover, J. W. | Blackman's al | Pittston | * | ...... |
| Hope, Christopher | ...... | Carnegie | * | ...... |
| Horner, John T. | ...... | Ohl | ...... | ...... |
| Hoskins, W. Horace | 3452 Ludlow st | Philadelphia | American Vet., 1881 | Philadelphia, 1889 |
| Hossler, Fred. B. | ...... | Hamburg | * | ...... |
| Hougendobler, J. J. | 1425 Derry st | Harrisburg | Ontario Vet., 1889 | Lancaster, 1889 |
| Hough, Peter | ...... | Perry | * | ...... |
| Houghton, Thos. J. | ...... | Lancaster | * | ...... |
| Houldsworth, J. D. | 163 Green lane | Manayunk | * | ...... |
| Houpt, S. | 20 W. Sunbury | Shamokin | * | ...... |
| Howett, David G. | 2245 N. 30th st | Philadelphia | * | ...... |
| Hoyt, W. T. | ...... | Hanover | * | ...... |
| Hubbard A. B. | 18 W. Central | Titusville | * | ...... |
| Huber, C. H. | ...... | Rural Valley | * | ...... |
| Hudson, E. B. | ...... | Sedgwick | * | ...... |
| Hufford, Samuel | ...... | Farmersville | * | ...... |
| Hughes, Wm. | ...... | Harrisburg | * | ...... |
| Huidekoper, Rush S. | 1304 Sansom st | Philadelphia | M.D. Univ. Pa., 1877<br>Alfort, France, 1892 | Philadelphia, 1889 |
| Hummel, Benj. | ...... | Mt. Pl's't Mills | * | ...... |
| Hummel, John | ...... | Knox | * | ...... |
| Hummel, R. A. | 917 N. 26th st | Philadelphia | ...... | Philadelphia, 1889 |
| Hummel, W. A. | ...... | Kratzerville | * | ...... |
| Humphrey, C. H. | ...... | Bethlehem | * | ...... |
| Husted, Ashley | ...... | Covington | * | ...... |
| Huyett, Walter G. | ...... | Wernersville | McKillip Vet., 1899 | Berks, 1899 |

(*To be continued.*)

